# Endoplasmic reticulum stress induces hepatic plasminogen activator inhibitor 1 in murine nonalcoholic steatohepatitis

**DOI:** 10.1096/fba.2020-00056

**Published:** 2020-10-10

**Authors:** Shantel Olivares, Anne S. Henkel

**Affiliations:** ^1^ Department of Medicine Northwestern University Chicago IL USA; ^2^ Jesse Brown VA Medical Center Chicago IL USA

**Keywords:** nonalcoholic fatty liver disease, unfolded protein response, X‐box binding protein 1

## Abstract

Plasminogen activator inhibitor 1 (PAI‐1) is a stress‐responsive gene that is highly induced in nonalcoholic steatohepatitis (NASH). Endoplasmic reticulum (ER) stress is a salient feature of NASH, yet it is unknown whether ER stress contributes to hepatic PAI‐1 induction in this disorder. Therefore, we aimed to (a) establish the role of ER stress in the regulation of hepatic *Pai*‐*1* expression, and (b) determine whether induction of *Pai*‐*1* in murine NASH is driven by ER stress. Hepatic *Pai*‐*1* expression was measured in C57BL/6 J mice and human HepG2 cells subjected to acute or prolonged pharmacologic ER stress. We found that hepatic *Pai*‐*1* expression was acutely suppressed in murine liver in response to severe ER stress followed by marked induction during the recovery phase of the ER stress response. Hepatic *Pai*‐*1* expression was induced in response to prolonged low‐grade ER stress in mice. Induction of *PAI*‐*1* by ER stress in HepG2 cells was prevented by pharmacologic inhibition of MEK1/ERK signaling or by siRNA‐mediated knockdown of *XBP1*, mediators of the recovery response to ER stress. Inhibiting ER stress with 4‐phenylbutyric acid prevented hepatic *Pai*‐*1* induction in mice with diet‐induced steatohepatitis. We conclude that hepatic *Pai*‐*1* is induced by ER stress via a pathway involving XBP1 and MEK1/ERK signaling, and induction of hepatic *Pai*‐*1* in murine NASH is mediated by ER stress. These data implicate ER stress as a novel mechanistic link between *Pai*‐*1* induction and NASH.

**Abbreviations**
4‐PBA: 4‐phenylbutyric acid
ER: endoplasmic reticulum
IL‐1β: interleukin 1β
JNK: cJun‐N‐terminal kinase
MCD: methionine and choline deficient
MCS: methionine‐ and choline‐sufficient
NAFLD: nonalcoholic fatty liver disease
NASH: nonalcoholic steatohepatitis
PAI‐1: Plasminogen activator inhibitor 1
Tgf‐β: transforming growth factor β
Tnfα: tumor necrosis factor α
XBP1: X‐box binding protein 1

## 1 INTRODUCTION

The endoplasmic reticulum (ER) functions to maintain protein homeostasis by regulating protein synthesis, folding and processing. Normal ER protein folding capacity can become impaired in response to various types of cellular stress, collectively termed “ER stress”.^1^ The resulting accumulation of unfolded or misfolded proteins triggers a highly evolutionarily conserved intracellular signal transduction pathway known as the unfolded protein response (UPR).^2^ The primary function of the UPR is to reduce protein burden and promote cell survival. If, however, these compensatory mechanisms are unable to adequately restore homeostasis, pathways leading to inflammation and apoptosis are initiated.^3^, ^4^ Although initially characterized for its role in maintaining protein homeostasis, the UPR is now recognized to regulate a myriad a cellular processes that impact a wide range of human diseases. In particular, UPR dysfunction has been strongly linked to human metabolic diseases such as obesity, diabetes, and nonalcoholic fatty liver disease (NAFLD).^5^, ^6^, ^7^, ^8^, ^9^, ^10^, ^11^, ^12^, ^13^

Plasminogen activator inhibitor‐1 (PAI‐1) is a stress‐responsive gene that is in induced in response to tissue injury and serves a physiologic role in wound healing. Although best known for its function as an inhibitor of fibrinolysis, PAI‐1 also regulates inflammation and fibrosis in numerous tissue types.^14^, ^15^, ^16^ In the liver, *PAI*‐*1* expression is low at baseline but becomes highly induced in response to numerous extracellular stimuli, consistent with its role as an acute phase protein. Classically, oxidative stress is a well‐established inducer of PAI‐1, however, other types of cellular stress have been implicated as well. Moreover, a host of inflammatory cytokines and growth factors, such as tumor necrosis factor α (Tnfα), interleukin 1β (IL‐1β), and transforming growth factor β (Tgf‐β), have been shown to induce PAI‐1 which, in turn, contributes to the cellular response to tissue injury.^17^, ^18^, ^19^, ^20^ Although *PAI*‐*1* is considered a stress responsive gene, the role of ER stress is the regulation of *PAI*‐*1* is unknown.

Beyond its role in fibrinolysis and wound healing, *PAI*‐*1* induction is increasing recognized as a feature of the human metabolic syndrome.^10^, ^11^, ^12^, ^13^ In particular, *PAI*‐*1* expression is highly induced in the livers of mice and humans with nonalcoholic steatohepatitis (NASH).^7^, ^8^, ^9^, ^21^ Although the association between metabolic disease and induction of *PAI*‐*1* is well‐established, the mechanism by which metabolic derangements induce *PAI*‐*1* is unknown. Given that induction of hepatic ER stress is a well‐recognized feature of NASH,^22^ we considered whether ER stress contributes to the characteristic induction of *PAI*‐*1* in NASH. Therefore, in the present study we aim to (a) determine the role of ER stress in the regulation of hepatic *Pai*‐*1* expression and (b) determine whether induction of *Pai*‐*1* in a murine model of steatohepatitis is regulated by ER stress.

## 2 MATERIALS AND METHODS:

### 2.1 Cell culture

Human hepatocellular carcinoma (HepG2) cells (ATCC, Mannasas, VA) were cultured in DMEM with 10% fetal bovine serum and maintained at 37°C in 5% CO_2_. Cells were grown to 80% confluence in six‐well plates and treated with 12 µmol/L tunicamycin, 100 nmol/L thapsigargin (Sigma‐Aldrich), 5 mmol/L DL‐homocysteine (Sigma‐Aldrich) or vehicle (DMSO/saline) in serum‐free DMEM for 6 or 18 hours. RNA isolation was performed using TRIzol Reagent (Invitrogen) per protocol. To determine whether the effects of tunicamycin are dependent on ERK1/2 signaling, HepG2 cells were treated with the MEK1 inhibitor, PD184352 (Santa Cruz Biotechnology) at a concentration of 1 µmol/L or vehicle (DMSO/saline). To determine whether the effects of tunicamycin are dependent on cJun‐N‐terminal kinase (JNK) signaling, HepG2 cells were treated with the JNK inhibitor, SP600125 (Sigma‐Aldrich) at a concentration of 25 µmol/L or vehicle (DMSO/saline). After one hour of exposure to PD184352 or SP600125, cells were treated with tunicamycin (10 µg/mL) (Sigma‐Aldrich) in serum‐free DMEM and incubated for an additional 6 hours. Cytotoxicity was assessed by measuring LDH release in cell culture media using a Cytotox 96 Nonradioactive Cytotoxicity Assay (Promega, Madison, WI). Human hepatoma Huh7 cells transfected with short hairpin RNA (shRNA) targeting XBP1 (Huh7^shXBP1^) or control shRNA (Huh7^shCON^) were generated and characterized as previously described ^23^ (kindly provided by Dr. Richard Green, Northwestern University). Huh7^shCON^ and Huh7^shXBP1^ cells were cultured in Dulbecco's modified Eagle's medium (DMEM) containing 10% fetal bovine serum, L‐glutamine, and penicillin‐streptomycin at 37°C with 5% CO2. To induce ER stress, cells were grown to 80% confluence in six‐well plates and treated with tunicamycin (10 µg/mL) or vehicle (DMSO/saline) in serum‐free media for 6 hours.

### 2.2 Animals and treatments

Wild‐type C57BL/6J male mice (Jackson Laboratories) at 8 weeks of age were treated with a single intraperitoneal injection of tunicamycin (2.0 mg/kg) or vehicle (20% DMSO/PBS) and sacrificed 6 or 72 hours post‐injection. To induce prolonged low‐grade ER stress, mice were treated with a cumulative dose of 0.5 mg/kg, 1.0 mg/kg, or 2.0 mg/kg tunicamycin or vehicle (10% DMSO) over 5 days. To determine the role of *Xbp1* in the regulation of *Pai*‐*1* in response to ER stress, mice bearing a hepatocyte‐specific deletion of Xbp1s were treated with a single injection of tunicamycin 0.5 mg/kg and sacrificed 3 days later. To determine the role of ER stress in mediating *Pai*‐*1* induction in a murine model of NASH, male C57BL/6 J mice (8‐10 weeks of age) were fed a methionine‐ and choline‐deficient (MCD) or methionine‐ and choline‐sufficient (MCS) diet and treated with either 4‐phenylbutyric acid (4‐PBA, 200 mg/kg/d I.P.) or vehicle (sterile saline I.P.) daily for 14 days. At the end of the treatment protocols, mice were sacrificed by CO2 inhalation followed by cardiac puncture. The collected blood was immediately centrifuged to collect the plasma. The livers were rapidly excised, flushed with ice‐cold saline, and sectioned. An aliquot was fixed in 10% formalin for histologic analysis which was performed at the Northwestern University Mouse Histology and Phenotyping Laboratory (Chicago, IL). The remainder of the liver was sectioned and snap‐frozen in liquid nitrogen. In select experiments, the hepatocyte fraction was isolated from C57BL/6 J mice treated with tunicamycin. Briefly, the vena cava was cannulized and perfused with a medium containing HBSS and EDTA for 8 minutes followed by DMEM with collagenase for 8 minutes. The liver was then removed, transferred to a petri dish containing perfusion medium, minced, and filter through a cell strainer. The cell suspension was then centrifuged, supernatant discarded, and the sediment containing parenchymal hepatocytes is washed several times. RNA was then isolated from the hepatocyte fraction. All animal protocols were approved by the Northwestern University Animal Care and Use Committee.

### 2.3 Blood and tissue analysis

Plasma and hepatic levels of total PAI‐1 were measured using an ELISA assay per manufacturer's instructions (Molecular Innovations, Novi, MI). Liver samples were homogenized in Dulbecco's phosphate buffered saline for hepatic lipid analysis (100 mg liver tissue/1 mL). Triglyceride levels were measured in liver homogenate using an Infinity spectrophotometric assay per the manufacturer's protocol (Thermo Electron Corporation). Total lipoperoxides were measured as thiobarbituric acid‐reactive substances (TBARS) in liver homogenate using a Lipid Peroxidation (MDA) Assay Kit (Sigma) and normalized to total protein concentration. Plasma ALT was measured using a spectrophotometric assay as per the manufacturer's protocol (Teco Diagnostics, Anaheim, CA).

### 2.4 Analysis of gene expression and protein expression

Total RNA from murine liver and harvested HepG2 cells was isolated using TRIzol reagent and real‐time quantitative PCR was performed as described previously.^24^, ^25^ Total protein was isolated and western blotting was performed as described previously.^24^, ^25^ Protein detection was performed using polyclonal rabbit antibodies to total and phosphorylated JNK and ERK (Cell Signaling Technology). Bound antibody was detected using goat anti‐rabbit polyclonal HRP antibody (Cell Signaling Technology) and developed using ECL Western Blotting Substrate (Cell Signaling Technology). Representative Western blots of pooled samples are shown.

### 2.5 Statistical analysis

Data are presented as mean ±standard deviation (SD). Comparisons between groups were performed using Student's *t* test analysis.

## 3 RESULTS

### 3.1 *PAI*‐*1* is induced by ER stress in a human liver cell line

**FIGURE 1:**
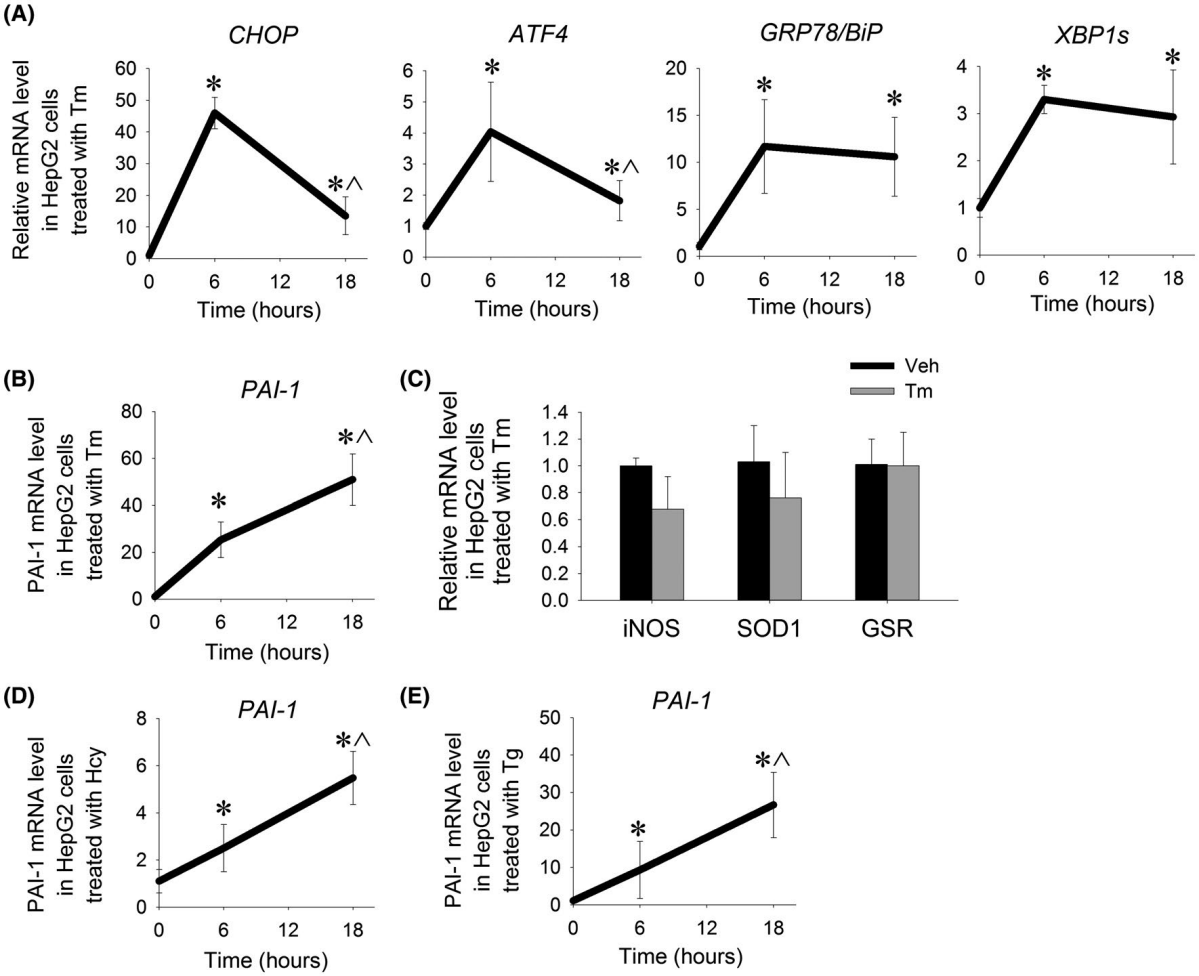
*PAI*‐*1* is induced by pharmacologic ER stress in a human liver cell line. Relative mRNA levels of A, ER stress markers and B, *PAI*‐*1* in HepG2 cells treated with 12 µmol/L tunicamycin (Tm) for 6 or 18 h. C, Relative mRNA levels of inflammatory and antioxidant markers in HepG2 cells treated with Tm for 6 hrs. D, *PAI*‐*1* mRNA level in HepG2 cells treated with 5 mmol/L DL‐homocysteine (Hcy) for 6 or 18 h. E, *PAI*‐*1* mRNA level in HepG2 cells treated with 100 nmol/L thapsigargin (Tg) for 6 or 18 h. *P < .05 vs vehicle‐treated cells, ^*P* <.05 vs 6 hr stressed cells

To determine the effect of ER stress on *PAI*‐*1* expression in a human liver cell line, HepG2 cells were treated with 12 µmol/L tunicamycin, a well‐established pharmacologic inducer of ER stress, for 6 or 18 hours. As expected, tunicamycin‐treated cells showed increased expression of *CHOP*, *ATF4*,*GRP78*/*BiP*, and *XBP1s* at 6 hours indicating robust UPR activation, followed by declining levels of *CHOP* and *ATF4* at 18 hours indicating resolving ER stress (Figure 1A). *PAI*‐*1* expression was induced in HepG2 cells at 6 hours after induction of ER stress (Figure 1B). Interestingly, the degree of *PAI*‐*1* induction increased at 18 hours, corresponding to resolving ER stress. Gene expression of iNOS, a well‐established marker of inflammation in HepG2 cells, and expression of antioxidant genes, superoxide dismutase 1 (SOD1) and glutathione reductase (GSR), showed no significant induction at the dose of tunicamycin used in these experiments, suggesting that the observed induction of *PAI*‐*1* is not the consequence of a nonspecific inflammatory response (Figure 1C). To ensure that the pattern of *PAI*‐*1* expression in response to ER stress is not specific to tunicamycin, HepG2 cells were treated with homocysteine and thapsigargin, two other pharmacologic agents that induce ER stress. Similar to the response observed with tunicamycin, treatment with either homocysteine or thapsigargin resulted in progressive induction of *PAI*‐*1* expression from 6 to 18 hours (Figure 1 D,E).

### 3.2 Induction of *PAI*‐*1* In response to ER stress is mediated by MEK1/ERK signaling

**FIGURE 2:**
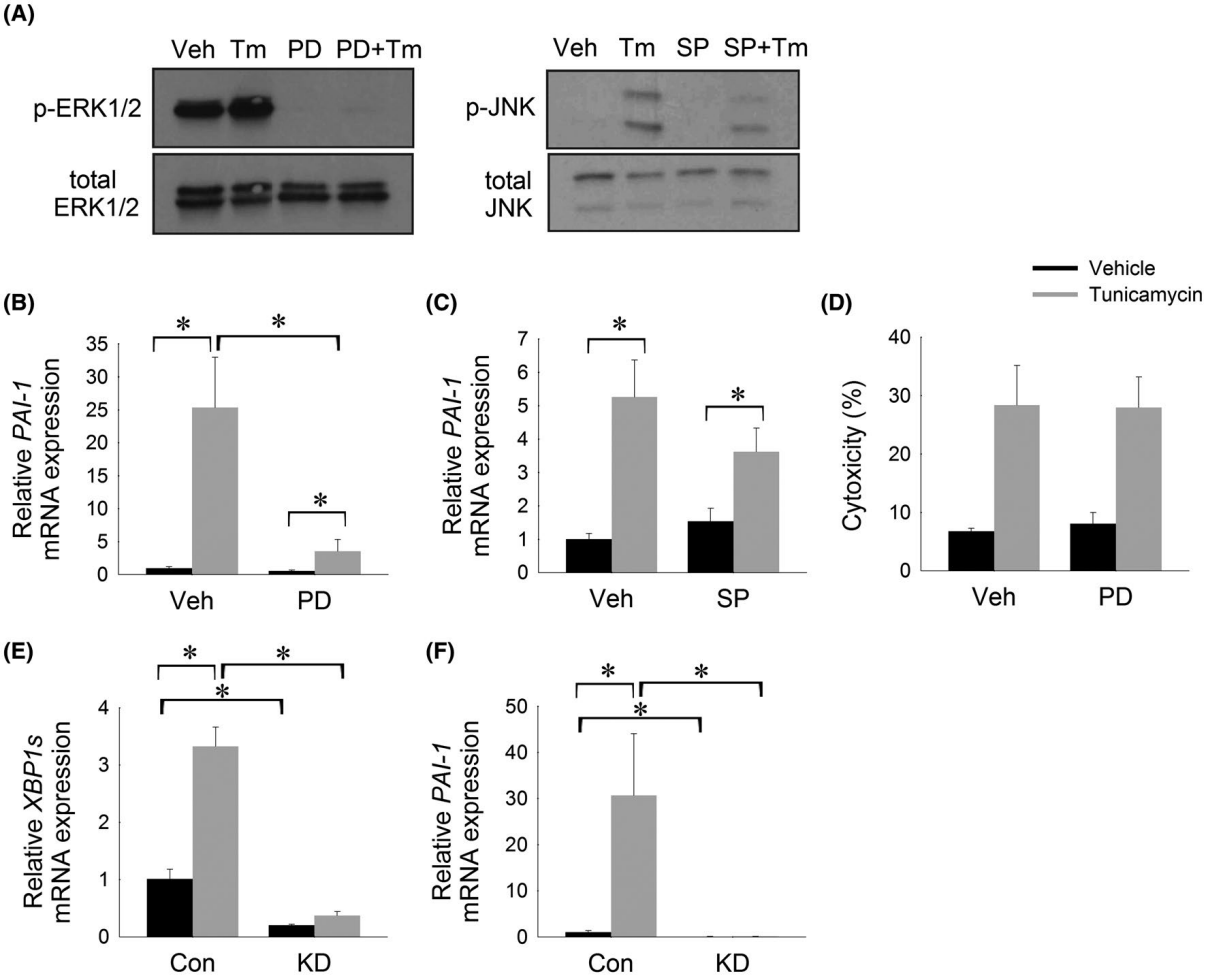
Induction of *PAI*‐*1* in response to ER stress is mediated by MEK1/ERK signaling and *XBP1*. A, Western blots demonstrating successful suppression of p‐ERK1/2 and p‐JNK by PD184352 (PD) and SP600125 (SP), respectively. Relative mRNA levels of *PAI*‐*1* in HepG2 cells treated pretreated with B, PD and C, SP followed by tunicamycin (10 µg/mL) for 6 h. D, LDH cytoxicity assay in HepG2 cells pretreated with PD followed by tunicamycin (10 µg/mL) for 6 h. Relative mRNA levels of E, *XBP1* and F, *PAI*‐*1* in Huh7 cells bearing a stable knockdown of *XBP1* (KD) or control (Con) cells treated with tunicamycin (10 µg/mL) for 6 h. *P < .05

Mitogen activated protein kinases (MAPKs), ERK and JNK, are activated in response to ER stress.^26^, ^27^, ^28^ Furthermore, ERK and JNK have both been shown to transcriptionally regulate *PAI*‐*1* and were, therefore, considered prime targets for mediating the effects of ER stress on *PAI*‐*1* expression.^18^, ^29^ HepG2 cells were treated with PD184352 or SP600125 to inhibit activation of ERK and JNK respectively, followed by treatment with tunicamycin for 6 hours. Successful inhibition of ERK and JNK activation by PD184352 and SP600125 was confirmed by western blot (Figure 2A). Pre‐treatment with a MEK1/ERK inhibitor significantly attenuated the degree of *PAI*‐*1* induction in response to ER stress (Figure 2B). In contrast, pre‐treatment with a JNK inhibitor, failed to significantly attenuate induction of *PAI*‐*1* (Figure 2C). As expected, treatment of HepG2 cells with tunicamycin was associated with increased cytoxicity as evidenced by increased LDH release compared to control cells (Figure 2D). Importantly, however, inhibition of ERK signaling did not attenuate this cytotoxicity, thus indicating that the attenuated *PAI*‐*1* induction in these cells is not due to attenuated cytotoxicity.

### 3.3 Induction of *PAI*‐*1* in response to ER stress is mediated by *XBP1*

Having shown that induction of PAI‐1 in response to ER stress is mediated, in part, via MEK1/ERK signaling cascade, we next sought to determine which branch of the UPR initiates this response. Induction of MEK1/ERK signaling in response to ER stress is thought to be a pro‐survival response aimed at counteracting ER stress.^26^, ^27^ The IRE1α‐XBP1 branch of the UPR controls the adaptive, pro‐survival function of the UPR. We therefore examined the effect of ER stress on *PAI*‐*1* expression in a human cell line with a stable knockdown of XBP1 (Huh7^shXBP1^). Huh7^shXBP1^ knockdown and Huh7^shCON^ control cells were treated with tunicamycin for 6 hours. Huh7^shCON^ control cells showed marked induction of both spliced (active) *XBP1 (XBP1s)* and *PAI*‐*1* in response to ER stress (Figure 2E,F). As expected, Huh7^shXBP1^ knockdown cells showed 80% suppression of *XBP1s* mRNA at baseline and no induction of *XBP1s* in response to ER stress (Figure 2E). Failure to induce *XBP1* splicing in Huh7^shXBP1^ cells resulted in a failure to induce *PAI*‐*1* expression (Figure 2F). These data indicate that *XBP1* mediates ER stress‐induced *PAI*‐*1* activation.

### 3.4 Hepatic *PAI*‐*1* is induced during the recovery from ER stress in mice

**FIGURE 3:**
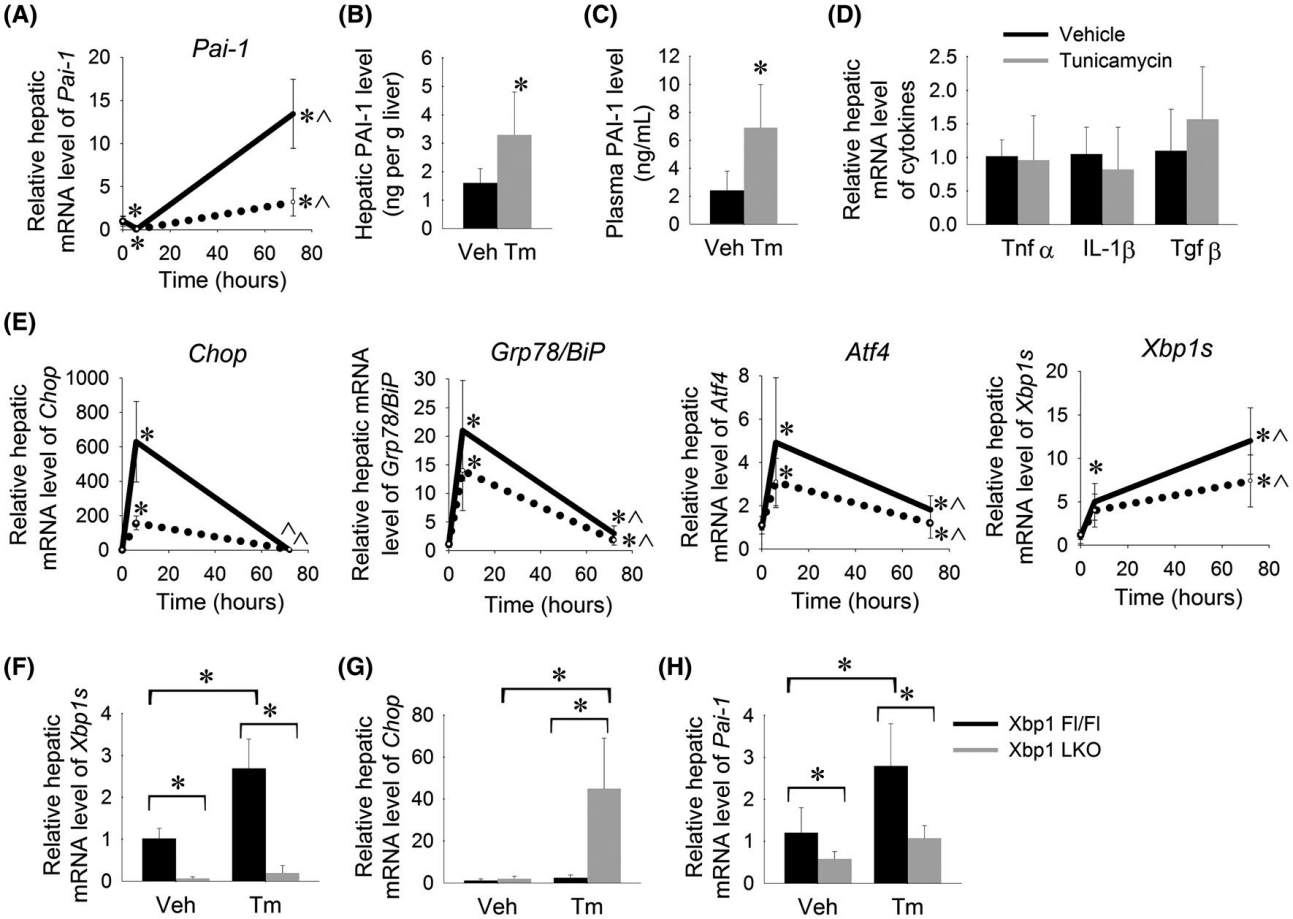
*Pai*‐*1* is an ER stress responsive gene in the liver. A, Hepatic mRNA expression of *Pai*‐*1*, B, hepatic protein level of PAI‐1 (ng per g liver), C, plasma PAI‐1 level (ng/mL), D, hepatic mRNA expression of pro‐inflammatory cytokines, and E, hepatic mRNA expression of ER stress markers were measured wild‐type C57BL/6J mice treated with a single I.P. injection of tunicamycin (solid line 2.0 mg/kg, dashed line 0.5 mg/kg) and sacrificed 6 or 72 h later. F, Relative hepatic mRNA level of F) *Xbp1s*, G, *Chop* and H, *Pai*‐*1* in *Xbp1^LKO^* and *Xbp1^fl^* ^/^ *^fl^* mice treated with tunicamycin 0.5 mg/kg I.P. and sacrificed 72 h later. *P < .05 vs vehicle‐treated mice, ^*P* < .05 vs 6 hr Tm‐treated mice

We next examined the effect of pharmacologic ER stress on hepatic *Pai*‐*1* expression *in vivo*. Wild‐type C57BL/6J mice were treated with a single intraperitoneal injection of 0.5 mg/kg or 2.0 mg/kg tunicamycin to induce hepatic ER stress. At either dose, hepatic expression of *Pai*‐*1* showed a biphasic response to ER stress (Figure 3A). In contrast to our observations in a human cell line, hepatic *Pai*‐*1* expression was significantly suppressed six hours after exposure to ER stress; however, by 72 hours, hepatic *Pai*‐*1* expression showed a ~4‐fold induction with low‐dose tunicamycin and ~15‐fold induction with high‐dose tunicamycin. Consistent with enhanced hepatic expression of *Pai*‐*1*, hepatic protein levels of PAI‐1 and plasma levels of PAI‐1 were increased at 72 hours after induction of ER stress (Figure 3B,C). It is well‐established that pro‐inflammatory cytokines induce *Pai*‐*1* expression^17^, ^18^, ^19^, ^20^; there was, however, no significant induction of *Tnfα*, *IL*‐*1β*, or *Tgfβ* in the liver to explain the increase in hepatic *Pai*‐*1* expression at 72 hours after induction of ER stress (Figure 3D).

To explore the mechanism of the biphasic response of hepatic *Pai*‐*1* to ER stress, we examined the pattern of UPR activation at 6 and 72 hours after induction of ER stress. Hepatic expression of *Chop*, *Grp78*/*BiP*,*Atf4*, and *Xbp1s* were all markedly induced by 6 hours, consistent with robust activation of the UPR (Figure 3E). By 72 hours, the time point at which hepatic *Pai*‐*1* expression was induced, hepatic expression of *Chop*, *Grp78*/*BiP*, and *Atf4* were nearing baseline consistent with resolving ER stress. Hepatic expression of *Xbp1s*, however, remained elevated consistent with ongoing activation of the pro‐survival elements of the UPR. These data indicate that hepatic *Pai*‐*1* is suppressed in response to acute ER stress followed by induction during the recovery phase of the UPR.

Having shown that *PAI*‐*1* induction in a human liver cell line is mediated, in part, via XBP1, we next examined whether this finding could be replicated in vivo. Mice bearing a liver‐specific deletion of *Xbp1* (*Xbp1* ^LKO^) and littermate controls (*Xbp1* ^fl/fl^) were treated with tunicamycin (0.5 mg/kg). As we have previously reported,^30^ *Xbp1* ^LKO^ fail to induce Xbp1 in response to ER stress and demonstrate failed suppression of *Chop* at 72 hours consistent with impaired capacity to resolve acute ER stress (Figure 3F,G). Consistent with an impaired recovery response to ER stress, *Xbp1* ^LKO^ mice demonstrated reduced induction of hepatic *Pai*‐*1* in response to ER stress (Figure 3H).

### 3.5 Prolonged ER stress induces hepatic *PAI*‐*1* expression

**FIGURE 4:**
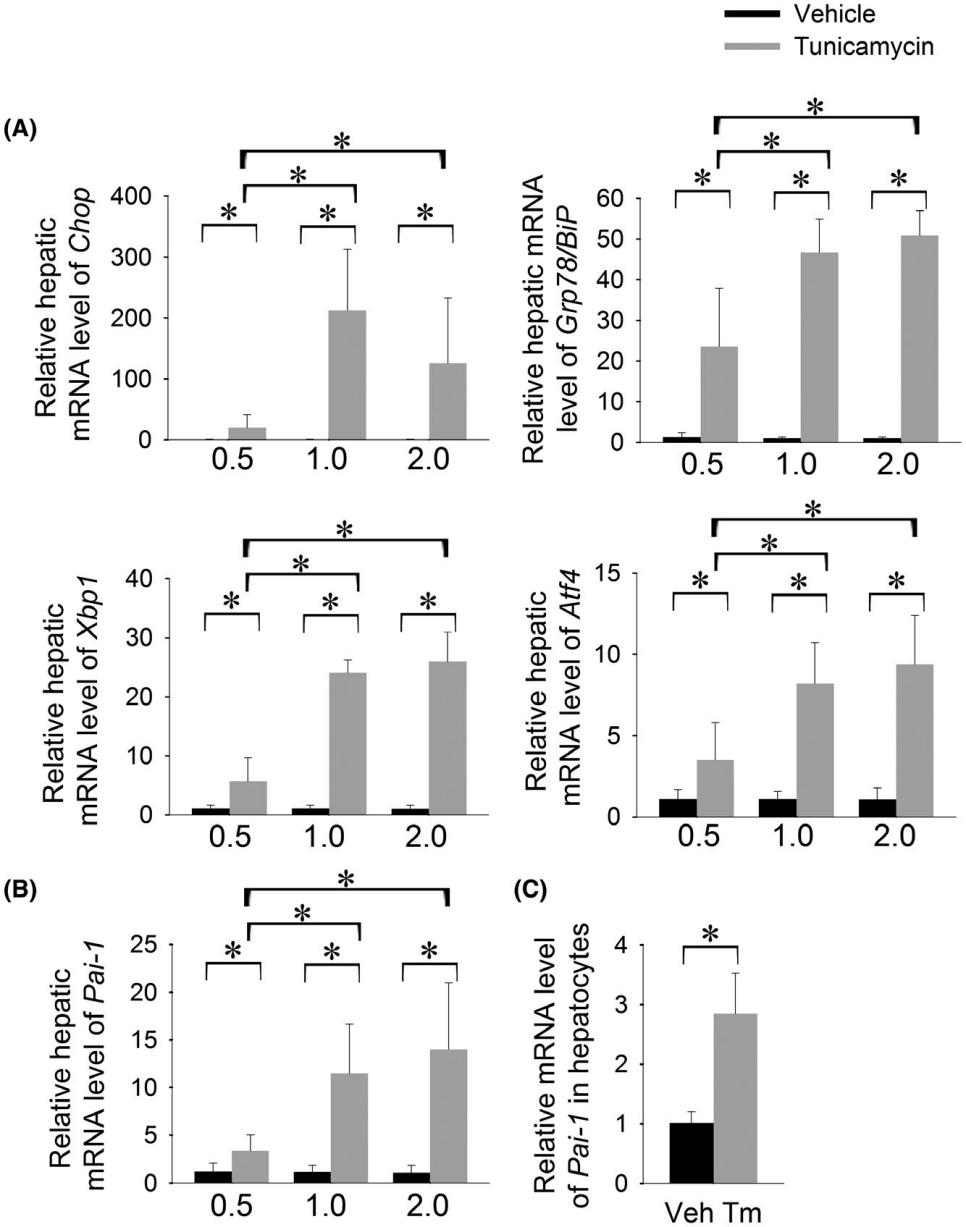
Hepatic *Pai*‐*1* expression is induced by prolonged low‐grade ER stress. Hepatic mRNA expression of A, ER stress markers and B, *Pai*‐*1* in wild‐type C57BL/6J mice treated with a cumulative dose of 0.5 mg/kg, 1.0 mg/kg, or 2.0 mg/kg tunicamycin over 5 d. C, Relative *Pai*‐*1* mRNA level in hepatocytes isolated from C57BL/6J mice treated with 0.5 mg/kg tunicamycin for 5 d. *P < .05

Numerous human diseases, including NASH, are associated with chronic low‐grade ER stress. We, therefore, determined the effect of prolonged, low‐grade ER stress on hepatic *Pai*‐*1* expression in mice. Mice were treated with a cumulative dose of 0.5 mg/kg, 1.0 mg/kg, or 2.0 mg/kg tunicamycin over five days. Tunicamycin‐treated mice demonstrated robust UPR activation that increased from the 0.5 mg/kg to the 1.0 mg/kg dosing and did not increase further with higher dosing (Figure 4A). Hepatic *Pai*‐*1* expression was induced 3‐fold in response 0.5 mg/kg tunicamycin and 11‐fold in response to 1.0 mg/kg (Figure 4B). The degree of Pai‐1 induction was similarly induced in response to the highest dose of tunicamycin, mirroring the plateau in UPR activation.

*Pai*‐*1* is expressed in both parenchymal and nonparenchymal liver cells raising the possibility that progressive induction of *Pai*‐*1* over time may be due to induction of *Pai*‐*1* in nonparenchymal cells as a reaction to liver injury. We therefore, determined whether the induction of *Pai*‐*1* in whole liver of mice treated with tunicamycin for 5 days could be recapitulated in the hepatocyte fraction. Hepatocytes isolated from tunicamycin‐treated mice showed induction of *Pai*‐*1* indicating that *Pai*‐*1* induction in whole liver is not driven exclusively by nonparenchymal cells (Figure 4C).

### 3.6 Inhibiting ER stress attenuates *PAI*‐*1* induction in a murine model of nonalcoholic steatohepatitis

**FIGURE 5:**
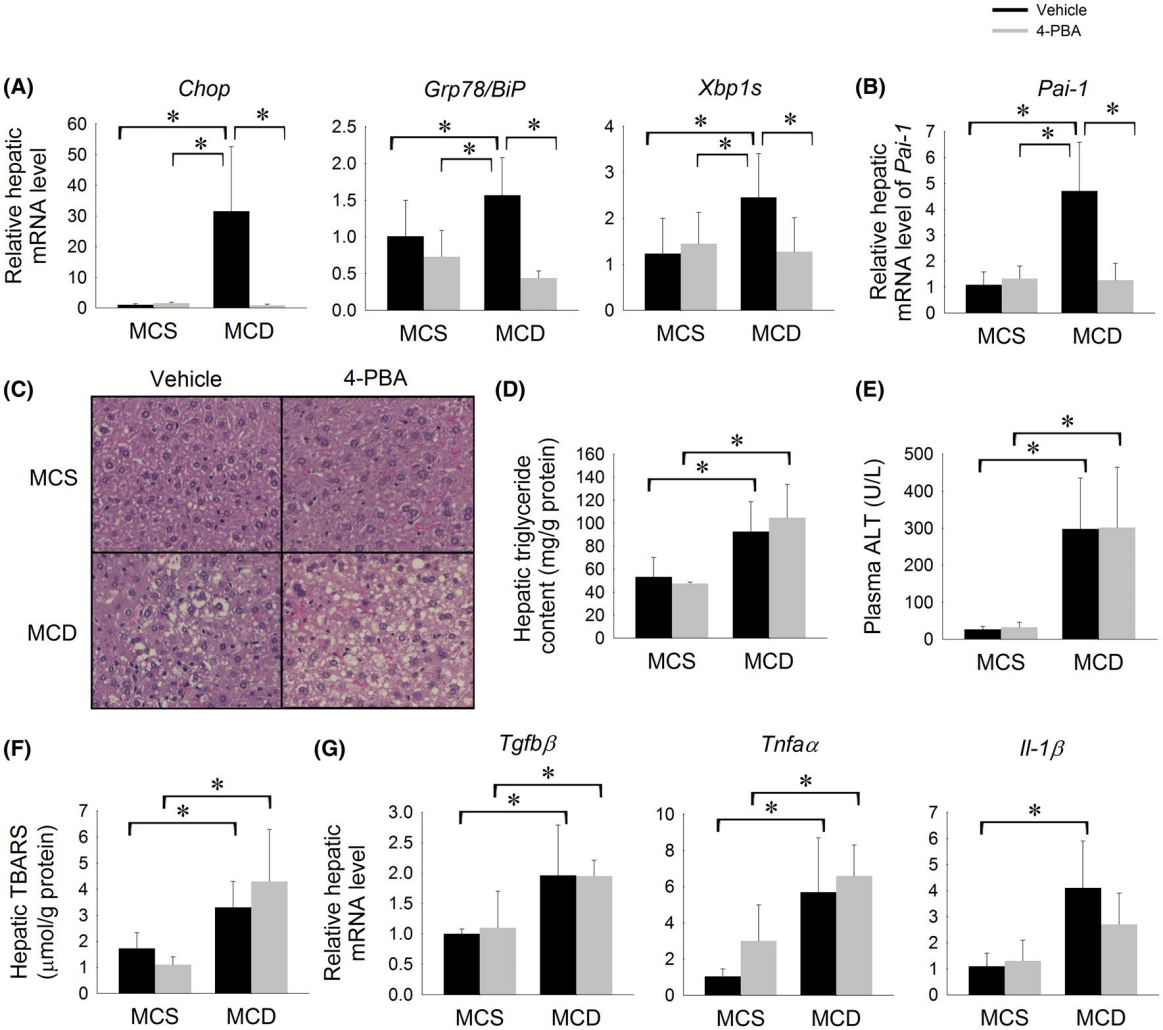
Inhibiting ER stress attenuates hepatic *Pai*‐*1* induction in mice fed a MCD diet. Relative hepatic mRNA expression of A, *Chop*, *Grp78*/*BiP*, *Xbp1s*, and B, *Pai*‐*1*, C, representative H&E stained liver sections, D, hepatic triglyceride content (mg trig/g protein), E, plasma ALT level (U/L), F, hepatic MDA level (µmol/g protein), and G, relative hepatic mRNA level of *Tgfβ*, *Tnfα*, and *IL*‐*1β* in C57BL/6J mice fed a control (MCS) or methionine‐ and choline‐deficient (MCD) diet with or without the ER chaperone, 4‐PBA, for 14 d. *P < .05

NASH is associated with chronic low‐grade hepatic ER stress as well as increased hepatic expression of *PAI*‐*1*. Having shown that hepatic *Pai*‐*1* is induced in response to prolonged low‐grade ER stress in mice, we next considered whether induction of *Pai*‐*1* in NASH is a consequence of hepatic ER stress. The methionine and choline deficient (MCD) diet is a well‐established murine model of progressive NASH. We have previously shown that MCD diet‐induced NASH is associated with marked induction of hepatic ER stress as well as increased hepatic expression of *Pai*‐*1*.^8^, ^31^ To establish whether this relationship is cause and effect, wild‐type C57BL6/J mice were fed a MCD or methionine‐ and choline‐sufficient (MCS) diet with or without the ER stress inhibitor, 4‐phenylbutyric acid (4‐PBA), for 14 days. Consistent with our previous observations in mice fed the MCD diet for 8 weeks, the livers of mice fed a MCD diet for 14 days showed activation of the UPR and significant induction of hepatic *Pai*‐*1* expression (Figure 5). Consistent with its established role as an inhibitor of ER stress, 4‐PBA suppressed the expression of *Chop*, *Xbp1s*, and *Grp78*/*BiP* in mice fed a MCD diet (Figure 5A). Inhibition of ER stress by 4‐PBA abolished the induction of hepatic *Pai*‐*1* expression observed in MCD diet‐fed mice (Figure 5B). Reducing ER stress in the MCD model did not attenuate hepatic lipid accumulation, lipid peroxidation, or hepatic inflammatory markers, indicating that the reduced induction of hepatic *Pai*‐*1* is not due to attenuated liver injury (Figure 5)C‐G). These data indicate that induction of hepatic *Pai*‐*1* in murine model of steatohepatitis is triggered by ER stress.

## 4 DISCUSSION

In the present manuscript, we demonstrate that *Pai*‐*1* is an ER stress‐responsive gene in the liver. Specifically, we find that hepatic *Pai*‐*1* expression is acutely suppressed in response to severe ER stress, followed by induction coinciding with the recovery phase of the UPR. Similarly, we show that prolonged low‐grade ER stress is associated with induction of hepatic *Pai*‐*1* expression in a dose‐dependent manner. Mechanistically, we find that activation of *PAI*‐*1* in response to ER stress occurs via a pathway involving *XBP1*, a critical mediator of the adaptive response to ER stress, and MEK1/ERK, a signaling cascade involved in counteracting ER stress.^26^, ^27^

Induction of PAI‐1 is a well‐established feature of NASH yet the mechanism by which PAI‐1 is induced in this disease has remained elusive. We demonstrate that inhibition of ER stress in a murine model of NASH prevents induction of hepatic *Pai*‐*1* expression. These data suggest that induction of hepatic *Pai*‐*1* in NASH may be driven by chronic low‐grade ER stress. This concept is in line with our previous work in which we examined the function of *Pai*‐*1* in MCD diet‐induced steatohepatitis.^8^ Specifically, we showed that induction of hepatic *Pai*‐*1* is a feature of MCD diet‐induced steatohepatitis, however, mice bearing a global deletion of *Pai*‐*1* (*Pai*‐*1* ^−/−^ mice) are not protected from MCD diet‐induced hepatic inflammation or fibrosis. As such, we concluded that induction of *Pai*‐*1* in this model may be a consequence, rather than a cause, of NASH. The present study supports the assertion that induction of *Pai*‐*1* is a component of the stress response initiated by NASH. Caution must be taken, however, in extrapolating mechanisms of MCD diet‐induced steatohepatitis in mice to human metabolic disease. The MCD diet produces histologic findings in the liver that closely recapitulate human NASH, however, the metabolic sequelae of MCD feeding do not parallel human disease. Additional work must be done to determine whether chronic low‐grade ER stress, a characteristic feature of the metabolic syndrome, drives *PAI*‐*1* induction in human obesity and its sequelae.

Although hepatic *Pai*‐*1* was induced in response to resolving acute ER stress and chronic low‐grade ER stress, paradoxically, its expression was markedly suppressed acutely in response to severe ER stress. The immediate goal of the UPR in response to severe, acute ER stress is to globally reduce protein synthesis. As a consequence, a host of genes affecting a myriad of cellular processes are acutely suppressed in response to severe ER stress. We suspect that the acute suppression of *Pai*‐*1* in response to severe ER stress may a component of this nonspecific response aimed at globally reducing cellular protein burden.

The delayed induction of hepatic *Pai*‐*1* after acute stress coincides with the recovery phase of the UPR, raising the possibility that *Pai*‐*1* is induced in response to tissue injury incited by severe ER stress. This would be in line with the known function of *Pai*‐*1* in responding to tissue injury and promoting wound healing. Arguing against this assertion, however, is the observation that there is a dissociation between ER stress, liver injury, and *Pai*‐*1* expression in the MCD model of NASH. Moreover, we have shown that attenuating ER stress with 4‐PBA does not reduce the severity of MCD diet‐induced hepatic steatosis, liver injury, or lipid peroxidation in mice; yet, attenuating ER stress in this model, did suppress hepatic *Pai*‐*1* induction indicating that induction of *Pai*‐*1* in this murine model of NASH is not purely a consequence of ER stress‐induced liver injury or oxidative stress. As such, we suspect that induction of hepatic *Pai*‐*1* in response to ER stress is not simply a nonspecific reaction to tissue injury but rather plays a specific role within the UPR in mediating the hepatic ER stress response. Further studies are warranted to determine the function of hepatic *Pai*‐*1* induction during the recovery from ER stress.

It is well‐established that *Pai*‐*1* is expressed not only by hepatocytes but also by nonparenchymal cells of the liver including endothelial cells, Kupffer cells, and hepatic stellate cells. We demonstrated that PAI‐1 is induced in HepG2 cells, a hepatocyte cell line, in response to ER stress. To confirm that this effect is not specific to an immortalized liver cell line, we also demonstrated that hepatocytes isolated from tunicamycin‐treated mice show upregulation of *Pai*‐*1*, indicating that induction of *Pai*‐*1* in murine liver in response to ER stress is not purely driven by nonparenchymal cells. However, it remains likely that multiple cells types within the liver contribute to the observed induction of *Pai*‐*1* in response to ER stress, and additional studies are warranted to explore the cell‐specific function of *Pai*‐*1* in NASH.

In summary, this work identifies a novel mechanism of *Pai*‐*1* regulation. Furthermore, we propose that ER stress may be the underlying mechanism for the previously unexplained induction of PAI‐1 that is characteristic of NASH. Based on these observations, additional studies are warranted to determine whether ER stress is a critical link between the metabolic syndrome and the increase in circulating PAI‐1 that is tightly associated with this spectrum of disorders.
